# Effect of remote ischemic conditioning on atrial fibrillation and outcome after coronary artery bypass grafting (RICO-trial)

**DOI:** 10.1186/1471-2253-11-11

**Published:** 2011-05-23

**Authors:** Daniel Brevoord, Markus W Hollmann, Stefan G De Hert, Eric HPA van Dongen, Bram GADH Heijnen, Anton de Bruin, Noortje Tolenaar, Wolfgang S Schlack, Nina C Weber, Marcel GW Dijkgraaf, Joris R de Groot, Bas AJM de Mol, Antoine HG Driessen, Mona Momeni, Patrick Wouters, Stefaan Bouchez, Jan Hofland, Christan Lüthen, Tanja A Meijer-Treschan, Benedikt H Pannen, Benedikt Preckel

**Affiliations:** 1 Department of Anaesthesiology, Laboratory of Experimental Intensive Care and Anaesthesiology (L.E.I.C.A.), Academic Medical Centre Amsterdam, The Netherlands; 2 Department of Anaesthesiology, St. Antonius Ziekenhuis, Nieuwegein, The Netherlands; 3 Clinical Research Unit, Academic Medical Centre Amsterdam, The Netherlands; 4 Department of Cardiology, Academic Medical Centre Amsterdam, The Netherlands; 5 Department of Cardiothoracic Surgery, Academic Medical Centre Amsterdam, The Netherlands; 6 Department of Anaesthesiology, UCL Brussels, Belgium; 7 Department of Anaesthesiology, UZ Gent, Belgium; 8 Department of Anaesthesiology, Erasmus Medical Centre Rotterdam, The Netherlands; 9 Department of Anaesthesiology and Intensive Care, University Hospital Düsseldorf, Germany

## Abstract

**Background:**

Pre- and postconditioning describe mechanisms whereby short ischemic periods protect an organ against a longer period of ischemia. Interestingly, short ischemic periods of a limb, in itself harmless, may increase the ischemia tolerance of remote organs, e.g. the heart (remote conditioning, RC). Although several studies have shown reduced biomarker release by RC, a reduction of complications and improvement of patient outcome still has to be demonstrated. Atrial fibrillation (AF) is one of the most common complications after coronary artery bypass graft surgery (CABG), affecting 27-46% of patients. It is associated with increased mortality, adverse cardiovascular events, and prolonged in-hospital stay. We hypothesize that remote ischemic pre- and/or post-conditioning reduce the incidence of AF following CABG, and improve patient outcome.

**Methods/design:**

This study is a randomized, controlled, patient and investigator blinded multicenter trial. Elective CABG patients are randomized to one of the following four groups: 1) control, 2) remote ischemic preconditioning, 3) remote ischemic postconditioning, or 4) remote ischemic pre- and postconditioning. Remote conditioning is applied at the arm by 3 cycles of 5 minutes of ischemia and reperfusion. Primary endpoint is the incidence AF in the first 72 hours after surgery, detected using a Holter-monitor. Secondary endpoints include length-of-stay on the intensive care unit and in-hospital, and the occurrence of major adverse cardiovascular events at 30 days, 3 months and 1 year.

Based on an expected incidence in the control group of 27%, 195 patients per group are needed to detect with 80% power a reduction by 45% following either pre- or postconditioning, while allowing for a 10% dropout and at an alpha of 0.05. With the combined intervention expected to be stronger, we need 75 patients in this group to detect a reduction in incidence of AF of 60%.

**Discussion:**

The RICO-trial (the effect of Remote Ischemic Conditioning on atrial fibrillation and Outcome) is a randomized controlled multicenter trial, designed to investigate whether remote ischemic pre- and/or post-conditioning of the arm reduce the incidence of AF following CABG surgery.

**Trial registration:**

ClinicalTrials.gov under NCT01107184.

## Background

It has been demonstrated previously that an organ can develop tolerance against ischemic stress by different interventions. In experimental studies, short periods (e.g., 3-5 minutes) of myocardial ischemia before the sustained ischemic period significantly reduced infarct size (early and late preconditioning) [1-3]. In addition, there is a significant amount of tissue that is damaged during early reperfusion, and it has been shown that staged reperfusion or short periods of ischemia (seconds to minutes) at the start of reperfusion, limits tissue damage (postconditioning) [4-6].

Besides periods of ischemia, several drugs may also induce this organ tolerance by pre- and postconditioning. These include nitrates, [7,8] opioids, [9,10] volatile anaesthetics [11-13] and noble gases [14,15]. While the application of short periods of organ ischemia is quite invasive and in most clinical settings not feasible, the named drugs mostly have significant hemodynamic or neurologic (anaesthetic) side effects preventing their use in a broad patient population (also outside the operating theatre).

Recently, a fascinating possibility to protect tissue against ischemia-reperfusion damage has been described. Remote ischemic pre- and postconditioning refer to the protective effect on the heart that can be induced by submitting another organ or skeletal muscle to multiple short periods of ischemia and reperfusion. Studies in animals have shown a reduction in infarct size after short periods of preconditioning ischemia (RIPC) on a remote organ [16,17]. In addition, clamping and releasing the femoral or renal artery before the onset of reperfusion can reduce myocardial infarct size (remote postconditioning, RpostC); [18] multiple cycles of ischemia reperfusion applied to the hind limb during myocardial reperfusion reduced infarct size in rabbits and pigs [19,20].

In humans, RIPC by short-term non-invasive limb ischemia reduced troponin I release after elective percutaneous coronary intervention (PCI) [21]. In patients subjected to coronary artery bypass graft (CABG) surgery, RIPC reduced myocardial damage as measured by biomarker release [22,23]. RpostC at the onset of reperfusion protected human endothelium against ischemia reperfusion injury [24]. Recently, this was translated to patients with acute myocardial infarction: applying short periods of forearm ischemia-reperfusion in patients with acute coronary syndrome by ambulance personal during transport to the hospital reduced infarct size as measured by cardiac perfusion scintigraphy [25].

Until today it is not known whether the reduction in tissue damage and reduced biomarker release will translate into better outcome of these patients. If an easily applicable procedure like remote conditioning leads to improved outcome in patients with planned or acute ischemic periods, such an intervention would be of great help in patient care. Hoole et al. demonstrated a reduction of major adverse cardiac events (MACE) after RIPC in patients undergoing elective PCI [21]. The role of RIPC and RpostC in the reduction of perioperative arrhythmias is unknown. Therefore, the present study investigates for the first time the long-time effects of RIPC, RpostC, and the combination of both procedures, in patients subjected to CABG surgery.

Atrial fibrillation (AF) is one of the most common complications after CABG surgery, affecting 27-46% of patients. It is associated with increased mortality, adverse cardiovascular events, and prolonged in-hospital stay. We hypothesize that RIPC and/or RpostC reduce the incidence of AF following CABG, and improve patient outcome.

## Methods/design

### Study objectives

The objective of this study is to investigate whether the safe and simple intervention of remote ischemic conditioning, (pre-, post-conditioning, or both), improves clinical outcome after CABG surgery, as measured by the incidence of postoperative atrial fibrillation, the length of stay on the intensive care unit (ICU) and in-hospital length of stay, and the occurrence of MACE at follow up.

### Study design

The RICO-trial is a randomized controlled multi-centre trial, utilizing four parallel arms. Patients are blinded for the treatment allocation since the study intervention will be done under general anaesthesia. In addition, investigators analyzing the data will be blinded and statistical analysis will be done using coded treatment groups, which will only be revealed after the analysis. However, local investigators responsible for inclusion of patients and executing the study protocol, will not be blinded

Patients scheduled for an elective, isolated CABG procedure, will be eligible. After inclusion, subjects will be randomized to one of the following arms 1) control, 2) remote ischemic preconditioning (RIPC), 3) remote ischemic postconditioning (RpostC), or 4) remote ischemic pre- and postconditioning (RIPC+RpostC). Randomization will be done using an internet based randomization application, with a biased coin approach and stratified for participating centre.

### Ethics

The study will be conducted in accordance with the principles of the Declaration of Helsinki, the Medical Research Involving Human Subjects Act (WMO) and the principles of "good clinical practice". The independent medical ethics committee of the Academic Medical Center in Amsterdam has approved the study for the hospitals in the Netherlands (approval number 09.017.1769, trial number MEC09/186), and the independent medical ethics committees of the centres in Belgium and Germany for these respective centres. Written informed consent is obtained from all participating patients.

The study is registered at ClinicalTrials.gov under NCT01107184.

### Participating centres

Patients will be enrolled in at least six centres; two academic centres and one non-academic centre in the Netherlands, two academic centres in Belgium and one academic centre in Germany.

### Study population

The study population consists of patients scheduled for elective CABG surgery in one of the participating centres.

Inclusion criteria are: elective CABG without valve surgery, the use of extra corporeal circulation, >18 years of age.

Exclusion criteria are: prior cardiac surgery, a history of AF, use of a class 1 or 3 anti arrhythmic drug or digoxin, intermittent aortic cross clamping during surgery, left ventricular ejection fraction ≤30%, serious pulmonary disease, renal failure, liver failure and the use of glibenclamide.

### Study outline

Local researchers will recruit and randomize patients, perform the study intervention, collect baseline, peri-operative and follow-up data and take care of the Holter-monitoring. Depending on the centre, patients will be screened and asked for consent either during the outpatient screening or while hospitalized prior to surgery. Randomization will be done after inclusion and prior to surgery.

All patients will have a surgical tourniquet placed on the upper arm prior to surgery. These tourniquets, which are also used in orthopaedic surgery to create a bloodless operating field, use compressed air and can create and maintain a preset pressure. Patients in the RIPC group and in the RIPC+RpostC group will have their tourniquet inflated to 200 mmHg for 3 × 5 minutes after the induction of anaesthesia but before the start of cardiopulmonary bypass. During aortic crossclamp, patients in both the RpostC group and the RIPC+RpostC group will have their tourniquet inflated for 3 × 5 minutes. In the control group, no tourniquet inflating will be done. Peripheral saturation and invasive blood pressure monitoring will be done using the contra-lateral arm, so that ischemic conditioning will not hinder monitoring. *Anaesthesia*

Anaesthesia will be performed according to good clinical standard procedures. Patients will be pre-medicated with midazolam 7.5 mg p.o. Induction of anaesthesia will be with intravenously (i.v.) applied midazolam 0,1-0,2 mg/kg and/or propofol TCI, i.v. sufentanil 1,0-1,5 μg/kg and i.v. rocuronium 0,6 mg/kg. Continuous infusion of sufentanil (0,3 μg/kg/h) and propofol TCI will be used for maintenance of anaesthesia; anaesthesia depth will not be monitored routinely.

#### Surgery

Surgical procedures include the use of extra corporal circulation but are otherwise not standardized, except for the exclusion of intermitted aortic cross clamping. Peri-operative care will be provided according to local standards.

#### Arrhythmias

In case AF develops, the patient should be treated according to local protocol, which may include either sotalol or amiodarone, but is not restricted to these medications. There will be no use of *prophylactic *medication for AF (e.g. prophylactic sotalol or amiodarone).

#### Postoperatively

At the end of surgery, prior to transportation to the intensive care unit, a Holter-monitor (Spacelabs Lifecard CF ECG) will be attached to the participants for monitoring of postoperative AF. After transportation to the ICU the monitor will be checked for proper attachment and thereafter once a day. After 72 hours the monitors will be collected and the data downloaded.

#### Follow-up

After discharge from the hospital, the patients will be contacted at 30 days, 3 months and 1 year after the initial surgery by phone and requested to fill out a mailed questionnaire. In addition, patients' physicians will be contacted after 1 year to complete the record. They will be questioned about the occurrence of rhythm disturbances, heart failure, revascularisation, acute coronary syndrome, myocardial infarction and transient ischemic attacks and stroke.

### Data collection and monitoring

Data of each patient will be noted in an individual case report form (CRF), identifiable by a study-specific patient identification number. CRF's will be stored locally and send electronically to the AMC. Holter data will be downloaded from the devices and transported or send to the AMC for central analysis and storage. Questionnaires will be mailed to the participants 30 days, 3 months and 1 year after surgery.

There will be regular meetings between the study coordinators and the main investigators of each site.

### Data analysis

Holter data analysis will be done using Spacelabs Pathfinder Software Kit (version 9.019) and under supervision of a cardiologist of the Academic Medical Centre Amsterdam, The Netherlands.

### Endpoints

#### Primary endpoint

The primary endpoint is the percentage of patients that develop AF during the first 72 hours postoperatively.

#### Secondary endpoint

Secondary endpoints include the percentage of patients with AF on each post-operative day, the total duration of AF and the number of episodes, the length of stay on the ICU as well as the in hospital stay and the MACE rate at 30 days, 3 months and 1 year follow-up.

### Statistical analysis

#### Sample size calculation

We hypothesize that pre- or post-conditioning will decrease the probability of AF by 40 to 50 per cent at a background rate of 27 per 100 CABG patients treated, which might be a clinically relevant reduction of AF. The effort of a combination of pre- and post-conditioning may only be relevant if there is additional reduction of AF, meaning that the combination is even more effective and might reduce the occurrence of AF by another 15%. In case of the pre- or post-conditioning groups a reduction in proportion AF from 0.27 to 0.1485 (minus 45%) seems feasible and clinically relevant. In case of the pre- and post-conditioning group a gain of at least 60%, from 0.27 to 0.108 (minus 60%), is considered clinically relevant. Sample sizes of 175 patients in the control group and 175 patients in the pre-conditioning group achieve 80% power to detect a difference between the group proportions of -0.1215. The test used is the two-group Chi^2^-test with a two-sided 0.05 significance level. Similarly, the number of patients sampled for the post-conditioning group should also be 175. With 175 patients in the control group, the number of patients needed in the pre- and post-conditioning group should be at least 66 to achieve 80% power to detect a difference between the group proportions of -0.162, using, again, a two-group Chi^2^-test with a two-sided 0.05 significance level. To account for 10% possible drop-out of patients 195 patients are needed in the control group, the pre-conditioning group, and the post-conditioning group respectively, while 75 patients are needed in the pre- and post-conditioning group. In total, 660 patients are needed.

## Discussion

Recently, remote ischemic conditioning gained attention as a possibility to protect different organs against ischemia reperfusion injury. Due to the safe and non-invasive nature of the intervention, this protection might be applicable in numerous patients. Small studies in cardiac surgery using biomarker release as endpoint suggest a beneficial effect of remote conditioning, [22,23,26-29] although not all studies are positive [30]. Although biomarker release following cardiac surgery is an accepted surrogate outcome parameter, [31-33] there is no evidence that remote conditioning leads to an improved outcome. Therefore, routinely use of remote conditioning cannot be recommended at this time. A study investigating the effects of remote ischemic conditioning on outcome parameters such as survival or quality of life would require a huge number of patients. We choose post-operative AF as a surrogate endpoint in this study, since it is not only a predictor of outcome, but prevention of AF also improves outcome [34-39]. The aetiology of AF following cardiac surgery is multi-factorial and not fully elucidated; however, ischemia reperfusion injury sustained during clamping of the aorta and generation of radical oxygen species during reperfusion play an important role. Ischemic conditioning is known to improve these processes. Indeed, direct ischemic conditioning has been shown to reduce post-operative arrhythmias [40-43]. Therefore, we hypothesize that remote ischemic conditioning can reduce AF.

Most ischemic periods (e.g., acute coronary syndrome, stroke) are unpredictable, and therefore RIPC is not feasible. A protective measure that can be applied after the onset of ischemia, such as RpostC, might have greater clinical implications. We therefore choose to include next to RIPC also RpostC, which has been demonstrated to be effective in animal and volunteer studies and a small trial in patients [18-20,24,44,45]. There is evidence that in aged or diseased myocardium, the ability to induce conditioning is reduced [46,46,47,49]. Therefore, we speculate that the added effect of both pre- and postconditioning could provide an additive protection not achieved by either intervention alone. Since such an effect should be more powerful to justify the extra effort, we expect an increased reduction in AF in the pre- and postconditioning group and will therefore include a smaller number of patients in this group.

In conclusion, the RICO-trial is a randomized controlled multicenter trial to investigate whether remote ischemic conditioning (RIPC, RpostC, or both) can reduce complications and improve outcome after CABG surgery.

## Competing interests

The authors declare that they have no competing interests.

## Authors' contributions

DB drafted the manuscript and BP co-authored the writing. MGWD provided statistical and methodological advice, JR de G will supervise the Holter-monitor data analysis. All other authors participated in the design of the study during several meetings and/or are local investigators in the participating centres. All authors have read and approved the final manuscript

## Pre-publication history

The pre-publication history for this paper can be accessed here:

http://www.biomedcentral.com/1471-2253/11/11/prepub
